# Increased Expression of *Oct4*, *Nanog* and *CD24* Predicts Poor Response to Chemo-Radiotherapy and Unfavourable Prognosis in Locally Advanced Oral Squamous Cell Carcinoma

**DOI:** 10.31557/APJCP.2020.21.9.2539

**Published:** 2020-09

**Authors:** Sridhar Mishra, Vandana Tiwari, Aditi Arora, Seema Gupta, Nidhi Anand, Nuzhat Husain

**Affiliations:** 1 *Department of Pathology, Dr. Ram Manohar Lohia Institute of Medical Sciences, Lucknow, Uttar Pradesh, India. *; 2 *Department of Radiotherapy, King George Medical University, Lucknow, Uttar Pradesh, India. *

**Keywords:** Oral squamous cell carcinoma, Oct4, Nanog, CD24, cancer stem cells

## Abstract

**Background:**

Current study investigates the role of *Oct4, Nanog *and *CD24* in locally-advanced oral squamous cell carcinoma (OSCC), to evaluate whether the expression of these markers can predict efficacy of neoadjuvant-chemo-radiotherapy and survival of patients.

**Methods:**

Biomarker expression was evaluated in 50 homogenously treated patients of locally-advanced OSCC.

**Results:**

Clinical response was complete in 30% (n=15), partial response in 46% (n=23), no response in 24% (n=12). Pathologically, 74% patents (n=37) were responders and 26% were non-responders (n=13). Biomarker-overexpression was seen in 46% cases for Oct4, 54% cases for Nanog and 58% cases for CD24. *Oct4, Nanog* and *CD24* expression showed significant correlation with clinical and pathological response (p<0.05). Three year recurrence-free survival was 71%, overall survival was 66%. Post-treatment advanced pathological N (ypN), post treatment advanced pathological TNM (ypTNM) stage, clinical non-response, pathologic non-response, positive/high expression of all three biomarkers had a significant negative impact on recurrence-free and overall survival.

**Conclusions:**

Expressions of *Oct4, Nanog* and *CD24 *have significant association with treatment response and survival in patients with locally advanced OSCC treated with neoadjuvant chemo-radiation. Survival of these patients is significantly affected by ypN stage, ypTNM stage, expression of all three biomarkers, clinical and pathological response to neoadjuvant therapy.

## Introduction

Oral Squamous cell carcinoma (OSCC) is a common malignancy in the oral cavity. Advances in the field of reconstructive surgery and diagnostic modalities had led to improvement in the survival of patients with OSCC. Local recurrence and distant metastasis occur. Unfortunately, advanced OSCC is refractory to treatment and leads to death in >50% of the cases (Jemal et al., 2011). Neoadjuvant chemo-radiotherapy is now an established modality for management of loco regional disease in patients with locally advanced operable OSCC (Kirita et al., 2012). Neoadjuvant chemo-radiotherapy helps in down-staging the primary tumor, making it resectable and eliminating micro metastases. Kirita et al., (2012), showed that preoperative cisplatin based chemotherapy and concurrent radiotherapy led to a clinical tumor response in 92.8% patients, with an overall 5-year survival of 79.3% in locally advanced resectable OSCC. However, all patients with advanced OSCC do not satisfactorily respond to neoadjuvant chemo-radiotherapy. It is hence critical to evaluate the molecular mechanisms for differential sensitivity to chemo-radiotherapy and detect molecular markers that can predict response.

It has been observed that cells that display stem cell-like characteristics within the tumor possess significant resistance to the current treatment modalities and promote tumor recurrence (Guo et al., 2014; Yanamoto et al., 2014). Obstinate properties of cancer stem cells (CSCs) against conventional chemotherapy regimens could explain anti-cancer therapy failure and recurrence in a number of cancer patients including those of OSCC in whom poor prognosis is related to the low response to chemotherapeutic drugs. Epithelial to mesenchymal transition (EMT) is a genetic hallmark of CSCs; change in tumor microenvironment stimulates EMT process that induces invasion and metastasis of tumors during cancer progression (Gupta et al., 2006). Thus understanding the role of CSCs in tumor initiation and progression has become a major focus in stem cell biology and cancer research. 

Oct4 (Pou5f1) is a transcription factor, which is strongly expressed in undifferentiated stem cells, which maintains pluripotency (Nichols et al., 1998; Pesce et al., 1998; Mitsui et al., 2003). Nanog, a downstream target of Oct4, is a homeodomain-containing protein which plays a key role in the maintenance, self-renewal and pluripotency of embryonic stem cells (Pesce et al., 1998; Mitsui et al., 2003). Inappropriate and untimely activation of Oct4 and Nanog result in CSCs instead of differentiated somatic or normal pluripotent stem cells. *CD24* biomarker expression is associated with aggressive tumours showing increase proliferative activity and invasion (Koukourakis et al., 2012; Kwon et al., 2007). CD24 is expressed in a variety of cancers and has been associated with shorter patient survival rates (Kristiansen et al., 2003a; 2003b; Sung et al., 2010; Choi et al., 2007; Chou et al., 2007; Sano et al., 2009)

In the current study, we analyzed the expression of and patterns of expression of *Oct4, Nanog* and *CD24* in a series of patients with locally advanced OSCC undergoing neoadjuvant chemo-radiotherapy, with the aim to evaluate whether the expression of these *CSC* markers correlate with efficacy of neoadjuvant chemo-radiotherapy response and survival in patients with OSCC.

## Materials and Methods

Patients: This study was conducted in the Department of Pathology, Dr. Ram Manohar Lohia Institute of Medical Sciences, Lucknow. Fifty patients of locally advanced OSCC, who were treated with neoadjuvant chemo-radiotherapy followed by surgical resection, were enrolled from a retrospective series. The inclusion criteria were (i) availability of a diagnostic biopsy in treatment naïve cases of OSCC, (ii) clinical stage III–IVa/b with no evidence of distant metastatic disease (M0), (iii) Patients who received completed regime of neoadjuvant chemo-radiotherapy and underwent surgical resection with curative intent, (iv) no other treatment given. The data of all patients was collected from medical records. Location of primary tumour and demographics of patients were recorded in all patients. The patients were staged clinically in preoperative phase and in pathological specimen postoperatively. retrospectively according to TNM classification proposed by American Joint Committee on Cancer (AJCC) (Amin and Edge 2017). The tumor grade was assigned according to World Health Organization classification (Pindborg et al., 2012) newer version available 

Treatment Protocol: All patients received chemo-radiotherapy by external beam conventional method (200cGy/fraction/day for 5 days a week) to a total dose of 70Gy in 35 fractions in 7-weeks to primary tumor site and neck along with concurrent weekly cisplatin (40mg/m^2^) (Gupta et al., 2009). Cisplatin was administered ambulatory with 1L intravenous hydration along with adequate antiemetic prophylaxis. Surgical resection was performed 4-6 weeks after the last dose of chemo-radiation and comprised of resection of primary tumour site with a margin of at least 1 cm along with neck node dissection (supraomohyoid neck dissection for clinically node negative and modified radical neck dissection for clinically positive nodes) as described by the American Head and Neck society (Robbins et al., 2002). 

Assessment of clinical response to neoadjuvant chemo-radiotherapy: The Response Evaluation Criteria in Solid Tumors (RECIST), version 1.1, was used to define objective soft-tissue response (Eisenhauer et al., 2009). Response to treatment of measurable lesions was assessed with contrast-enhanced computed tomography (CECT) scan 6 weeks after neoadjuvant chemo-radiotherapy.

• Complete Response (CR): “disappearance of all target lesions with any pathological lymph nodes must have reduction in short axis to<10 mm.”

• Partial Response (PR): “At least a 30% decrease in the sum of diameters of target lesions, taking as reference the baseline sum diameters.”

• Progressive Disease (PD): “At least a 20% increase in the sum of diameters of target lesions, taking as reference the smallest sum on study. In addition to the relative increase of 20%, the sum must also demonstrate an absolute increase of at least 5 mm.”

• Stable Disease (SD): “Neither sufficient shrinkage to qualify for PR nor sufficient increase to qualify for PD, taking as reference the smallest sum diameters while on study.”

Clinical response was categorized as complete responders, partial responders and non-responders. Non responders included patients with progressive disease. 

Assessment of pathologic response: It was evaluated on resection specimen using 4 grade of pathological regression (Braun et al., 1989)

• Regression grade 1 (RG1 or complete pathologic response): No residual viable tumour

• Regression grade 2 (RG2 or microscopic residual foci): less than 5% viable tumour

• Regression grade 3 (RG3): 5-50% viable tumour

• Regression grade 4 (RG3): more than 50% viable tumour

RG1 and RG2 were categorized as responders, while RG3 and RG4 as non-responders.

Immunohistochemistry: Formalin fixed paraffin embedded tumour blocks of pre-treatment biopsy sample of patients were used for Immuohistochemical (IHC) analysis for this study.Tissue sections of 5μm were deparaffinised in xylene and then re-hydrated with sequential washes of ethanol. Endogenous peroxidase activity was inhibited with 3% hydrogen peroxidase (Loba Chemie, India) in methanol for 30 minute. For antigen retrieval, slides were placed in 50 ml citrate buffer pH6.0 to unmask the epitopes. Tissue sections were then incubated with various antibodies. Anti-human antibodies Oct4 (Sigma, USA) used at a dilution of 1:100; Nanog (Thermo Scientific, USA) at a dilution of 1:75, and CD24 (Thermo Scientific, USA) in dilution of 1:25, were added for incubation followed by washing with wash buffer, three times followed by treatment with polymer based secondary antibody kit with 3′3 diaminobenzidine tetra hydrochloride (DAB), as substrate (DAKO, Denmark). All sections were counterstained with 0.1% haematoxylin and fixed with permanent mounting medium and covered with glass cover slips. Negative controls and recommended positive controls were used. The expression of stem cell markers was assessed as a percentage of positive tumours cells in hot spots (10 High Power Fields) as reported previously (Soni et al., 2014). Two pathologists (N.H, A.A) scored all samples blindly without knowing clinical characteristics and prognosis.

**Figure 1:**
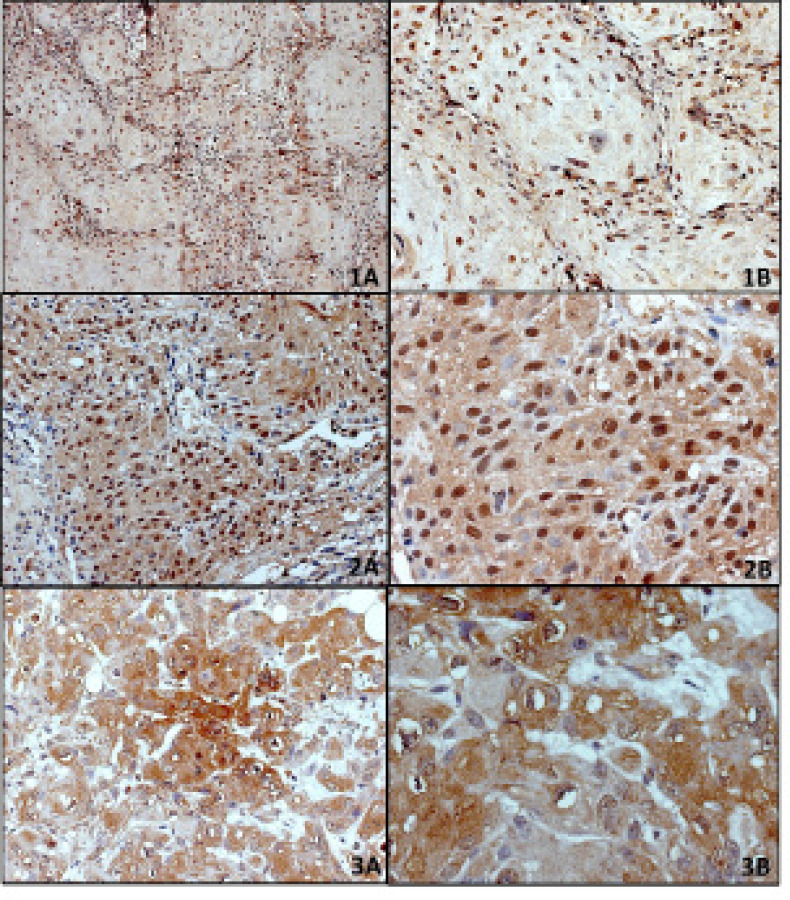
Micro Photograph Showing Immuohistochemical Staining for Oct4, Nanog and *CD24* Expressions in Oral Squamous Cell Carcinoma. High positive expression of *Oct4* in the nucleus of tumor cells (1a) 20X; (1b) 40X. High positive expression of Nanog in the nucleus of tumor cells (2a) 20X; (2b) 40X. High positive expressions of CD24 in the cytoplasm of tumor cells (3a) 20X; (3b) 40X. (DAB x 125 x digital magnification)

Evaluation of staining for *Oct-4, Nanog* and *CD24*: For evaluating expression of both *Oct4* and *Nanog* only nuclear staining was considered as positive. The membranous and the cytoplasmic staining of CD24 was evaluated separately, and cytoplasmic staining was considered positive. IHC results were assessed in terms of the proportion of tumor cell staining according to the previous published method with modifications.23 For statistical analyses, <10% expression was consider as negative and ≥10% as positive. Figure 1 shows representative examples of *Oct4, Nanog* and *CD24* immunohistochemistry. 

Outcomes: Variables assessed were age, sex, risk factors (including tobacco consumption, betel nut chewing, human papilloma virus infection, and alcohol consumption), location of tumor, grade of tumor, clinical N and TNM stage, pathologic N (ypN) and TNM stage (ypTNM) after neoadjuvant treatment post-treatment pathologic and clinical response, as well as the expression of* Oct4, Nanog* and *CD24*. Overall survival was the primary end point of our study. Secondary end points were response to treatment and recurrence-free survival. The time from surgery to death due to any cause was defined as overall survival. The time from surgery to recurrence of cancer (local or distant) or death without recurrence was defined as recurrence-free survival. 

Statistical Analysis: Statistical analysis was performed using the Statistical Package for Social Sciences (SPSS) analysis software version 20. The association of clinical and pathological response after neoadjuvant therapy with expression of biomarkers and clinico-pathological parameters was assessed using either Chi-square (*χ*^2^) methodor Fisher’s exact tests, as appropriate. Association of mean expression of biomarker with clinico-pathological parameters and type of response was assessed by Mann-Whitney U test; one way analysis of variance (ANOVA) and Kruskal Wallis test (for more than two groups, as appropriate). Cox proportional hazards regression analysis was performed to evaluate the effects of biomarkers and other clinico-pathological variables on recurrence free survival and overall survival. Statistical significance was defined as p-value <0.05. 

## Results

Characteristics of the patients: A total of 50 cases were enrolled in the study, which included 82% males (n=41) and 18% females (n=9) with age ranging from 25 to 75 years (49.3±11.3 years). The location of primary tumour was buccal mucosa in 42% (n=21), tongue in 28% (n=14), retro molar trigone in 10% (n=5), and other (cheek, palate and lip) in 20% (n=10). The baseline demographics and clinicopathological parameters have been summarised in Table 1. Clinical N stage was N0 in 22% (n=11), and N+ in 78% (n=39). Clinical TNM stage was III in 44% (n=22), and IVa/b in 56% (n=28).Pathological N stage after neoadjuvant therapy (ypN stage) was 0 in 70% (n=35), 1 in 24% (n=12) and 3 in 6% (n=3). Pathological TNM stage after neoadjuvant therapy (ypTNM stage) was 0:1:2:3:4 in 40 % (n=20): 18% (n=9): 12 % (n=6): 16% (n=8): 14 % (n=7) respectively.

Out of 50 patients, clinical response was complete in 30% (n=15), partial response in 46% (n=23), no response in 24% (stable disease in 7 and progressive disease in 5). Pathologically, 74% patents (n=37) were responders, and 26% were non-responders (n=13). Fifteen patients (30%) were classified as RG1, 22 (44%) as RG2, 7 (14 %) as RG3 and 6 (12%) as RG4. Follow-up data were available for all 50 patients. At follow-up of 36 months, 17 patients (34%) had died: 10 owing to tumor recurrence and 7 owing to other causes. Of the 33 surviving patients (66%), two were alive with tumor recurrence, and 31 were free of tumor. Of all 50 patients, 12 (16%) developed tumor recurrence (8 local, 2 regional, and 2 distant metastasis).

Association of clinical and pathological response with qualitative biomarker expression and clinico-pathologic parameters: Based on the chosen cut off levels (>10%), over expression was seen in 23 of 50 (46%) cases for Oct4, in 27 (54%) for Nanog and in 29 (58%) for CD24. A statistically significant association was found between negative expression (<10%) of *Oct4, Nanog, CD24* and clinical response (*χ*^2^ =13.28, p value = 0.001 for Oct4, *χ*^2^ = 15.30, p value = 0.0001 for Nanog and *χ*^2^ =7.93, p value = 0.01 for CD24). Similarly, a statistically significant association was found between negative expression (<10%) of *Oct4, Nanog, CD24* and pathological response (*χ*^2^ =8.55, p value = 0.003 for Oct4, *χ*^2^ = 5.07, p value = 0.02 for Nanog and *χ*^2^ =2.38, p value = 0.03 for CD24). The relationship between biomarker expression and response has been summarised in Table 2. 

Association of quantitative expression of biomarkers with demographic characteristics and response to neo-adjuvant therapy: Significant difference was observed in the mean expression of all three biomarkers among pathological responders and non-responders (17.32**±**6.83 vs. 37.15**±**13.65 for Oct4; 18.86**±**10.21 vs. 39.92**±** 13.93 for Nanog; 22.37**±**12.26 vs. 47.93**±**13.57 for CD24) and p value was <0.05 as summarized in Table 3. A significant difference was observed in expression of all three biomarkers among patients in whom clinical response were complete, partial and non-responders (12.67**±**4.31 vs. 21.41**±**9.38 vs. 39.01**±**14.74 for Oct4, p<0.0001; 11.57 **±**5.93 vs. 20.98**±**10.13 vs. 38.83**±** 16.35 for Nanog, p<0.0001; 16.76**±** 9.54 vs. 29.93**±**14.02 vs. 48.81**±**15.34 for CD24, p<0.0001). 

Survival analysis: Recurrence-free survival was 71% at 3 years. In Cox regression analysis, advanced ypTNM stage (P =0.03), clinical non-response (p=0.006), pathologic non-response (P< 0.002), positive expression of all three biomarkers (dichotomized variable, p=0.0001) and high expression of all three biomarkers (continuous variable, p=0.0001) had a significant negative effect on recurrence-free survival as shown in Table 4. The overall survival rate of all 50 patients was 66% at 3 years. Cox regression analyses showed that advanced ypN stage (P=0.02), advanced ypTNM stage (P=0.01), clinical non-response (p=0.04), pathologic non-response (P=0.008), positive expression of all three biomarkers (dichotomized variable, p=0.0001) and high expression of all three biomarkers (continuous variable, p=0.0001) were significantly associated with decreased overall survival. 

**Figure 2:**
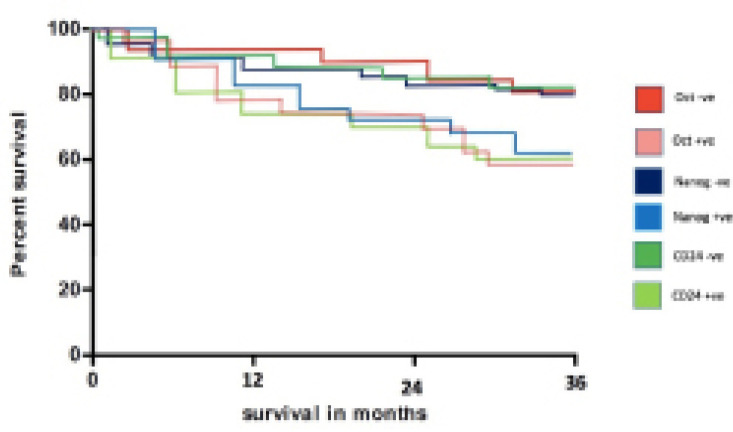
Kaplan Meier Graphs Showing the Cumulative 3-Year Survival of Patients According to Immunoreactivity for Oct4, Nanog and CD24 which was 59% for Oct4 +ve, 62% for Nanog +ve and 60% for CD24 +ve as compared to 81.0% for Oct4 -ve, 79% for Nanog –ve and 82 % for CD24 –ve respectively (Log-rank test, P<0.05)

Survival rates were compared between clinical outcome of neoadjuvant chemoradiation and expression of CSC markers using Kaplan-Meier method. Patients with overexpression of markers had a significantly unfavorable outcome compared to those with negative expression (Log-rank test, p<0.05) (Figure 2). The cumulative survival rate for 3 years in the positive expression group was 59% (*Oct4*), 62% (*Nanog*) and 60% (*CD24*) whereas that in the negative expression groups was 81% (*Oct4*), 79% (*Nanog*) and 82% (*CD24*). 

**Table 1:** Demographic and Clinico-Pathological Characteristics of Patients

Characteristics	Patient n= 50 (%)
Age	
25-50 yrs	30 (60)
>50 yrs	20 (40)
Sex	
Male	41 (82)
Female	09 (18)
Tobacco consumption	
Chewing	41 (82)
Smoking	21 (42)
Betel nut chewing	30 (60)
Alcohol intake	13 (26)
Site of primary tumour	
Buccal Mucosa	21 (42)
Tongue	14 (28)
Retro moral trigone	05 (10)
Cheek	04 (8)
Palate	03 (6)
Lip	03 (6)
Histological grading	
Well Differentiated	09 (18)
Moderately differentiated	36 (72)
Poorly differentiated	05 (10)
Clinical N stage	
N0	11 (22)
N+	39 (78)
Clinical TNM stage	
Stage III	22 (44)
Stage IVa/b	28 (56)
ypN stage*	
0	35 (70)
1	12 (24)
2	03 (6)
Yp TNM stage **	
0	20 (40)
1	09 (18)
2	06 (12)
3	08 (16)
4	07 (14)
Clinical response	
Complete Responder	15 (30 )
Partial Responder	23 (46)
Stable disease	07 (14)
Progressive disease	05 (10)
Pathological response	
RG1	15(30)
RG2	22 (44)
RG3	07 (14)
RG4	06 (12)

*ypN stage, pathologic N stage after neoadjuvant therapy; ** ypTNM stage, pathologic TNM stage after neoadjuvant therapy

**Table 2:** The Association of Clinical and Pathological Response after Neoadjuvant Therapy with Expression of the Biomarkers and Clinic-Pathological Parameters

	No. of cases (%)	Clinical Response				Pathological Response		
Parameters		Complete response	Partial response	No response	p-value	Response	No response	p-value
*Oct4* Expression								
Positive	23 (46)	5	7	11	0.001	12	11	0.003
Negative	27 (54)	10	16	1		25	2	
Nanog Expression								
Positive	27 (54)	2	15	10	0.0001	16	11	0.02
Negative	23 (46)	13	8	2		21	2	
*CD 24* Expression								
Positive	29 (58)	2	16	11	0.01	17	12	0.03
Negative	21 (42)	12	7	2		20	1	
Age:								
25-50 yrs	30 (60)	10	15	5	0.33	23	7	0.84
>50 yrs	20 (40)	5	8	7		14	6	
Sex								
Male	41 (82)	11	20	10	0.56	31	10	0.89
Female	09 (18)	4	3	2		6	3	
Grade								
WD	09 (18)	3	4	2		7	2	
MD	36 (72)	10	17	9	0.98	26	10	0.9
PD	05 (10)	2	2	1		4	1	
Clinical N stage								
N0	11 (22)	4	5	2	0.83	7	4	0.61
N+	39 (78)	11	18	10		30	9	
Clinical TNM stage								
Stage III	22 (44)	6	12	4	0.52	16	6	0.82
Stage 1Va/b	28 (56)	9	11	8		21	7	

**Table 3:** The Association of Expression of Biomarkers with Demographic and Response to Neo-Adjuvant Therapy

Parameter	*Oct-4* Expression	p value	*Nanog* Expression	P-value	*CD24* Expression	P-value
	(Mean ±SD)		(Mean ±SD)		(Mean ±SD)	
Age						
25-50 yrs	28.48 ± 12.15	0.125	27.22 ±11.96	0.494	41.37 ±13.81	0.665
>50 yrs	23.30± 10.43		25.02 ±9.54		39.70 ±12.42	
Sex						
Male	26.52 ±13.73	0.851	26.71±12.48	0.6003	38.44±14.80	0.918
Female	25.55 ±15.45		24.34±10.73		37.89 ±12.67	
Histological Grade						
WD	25.10±11.26		24.20 ±11.32		38.94±12.10	
MD	28.75 ±12.98	0.731	23.16±13.54	0.846	37.50 ±11.61	0.813
PD	27.16 ±10.89		26.75±14.46		40.81±10.43	
Clinical N Stage						
N0	23.41±11.17	0.835	28.61±13.40	0.521	37.51±11.75	0.629
N	22.52±12.83		25.93±11.79		35.73 ±10.46	
Clinical TNM Stage						
III	26.29±12.14	0.881	26.68 ±12.74	0.851	37.45 ±14.47	0.841
IV	25.74±13.43		27.35 ±12.35		36.68±12.64	
Clinical Response:						
Complete Responders	12.67±4.31	<0.0001	11.57 ±5.93	<0.0001	16.76± 9.54	<0.0001
Partial Responders	21.41 ±9.38		20.98 ±10.13		29.93 ±14.02	
Non Responders	39.01±14.74		38.83 ±16.35		48.81±15.34	
Histological Response						
Response (R1/R2)	17.32±6.83	0.0001	18.86±10.21	0.0001	22.37±12.26	0.0001
No response (R3/R4)	37.15±13.65		39.92±13.93		47.93±13.57	

**Table 4:** Cox Regression Analyses of Recurrence-Free Survival and Overall Survival

	Recurrence-free survival			Overall survival		
	Hazard ratio	95% CI	p value	Hazard ratio	95% CI	P-value
Age	1.18	0.63-3.21	0.87	1.43	0.82-3.14	0.32
Sex	0.92	0.56-1.67	0.65	0.61	0.35-1.39	0.78
Tobacco consumption	1.08	0.73-1.75	0.76	1.12	0.72-1.84	0.63
Betel nut chewing	0.74	0.41-1.23	0.91	0.89	0.46-1.73	0.88
Alcohol intake	0.87	0.57-1.34	0.67	0.76	0.49-1.82	0.61
Site of primary tumour	0.67	0.39-1.42	0.29	0.72	0.35-1.57	0.58
Histological grading	1.52	0.77-2.31	0.76	1.41	0.68-2.78	0.49
Clinical N stage	1.74	0.41-2.57	0.42	1.53	0.72-2.41	0.16
Clinical TNM stage	1.27	0.32-2.62	0.32	1.47	0.58-2.94	0.37
ypN stage^a^	1.24	0.43-2.47	0.13	1.76	1.23-2.35	0.02
yp TNM stage^b^	1.42	1.12-2.53	0.03	1.76	1.49-2.83	0.01
Clinical response^c^	1.48	1.32-3.63	0.006	1.38	1.18-3.27	0.04
Pathological response^c^	1.52	1.24-3.11	0.002	1.46	1.02-3.71	0.008
Oct4 positive (>10%)	1.42	1.89-2.61	0.001	1.75	1.37-2.56	0.001
Oct4^d^	1.56	1.24-2.51	0.001	1.17	1.03-1.67	0.001
Nanog positive (>10%)	1.56	1.18-2.47	0.001	1.59	1.24-2.32	0.001
Nanog ^d^	1.11	1.79-2.81	0.001	1.71	1.12-2.39	0.001
CD 24 positive (>10%)	1.75	1.03-2.44	0.001	1.72	1.23-2.57	0.001
CD 24^d^	1.82	1.52-2.48	0.001	1.49	1.12-2.55	0.001

^a^ypN stage, pathologic N stage after neoadjuvant therapy; ^b^ypTNM stage, pathologic TNM stage after neoadjuvant therapy; ^c^Clinical and pathologic response was evaluated as described in Materials and methods; ^d^Continuous variable.

## Discussion

Cancer stem cells (CSCs) play an important role in initiation, propagation, metastasis, recurrence, and therapeutic failure of OSCC. The CSC theory hypothesises that unregulated asymmetric division of CSCs generate dissimilar population of differentiated progenitor cells that eventually make up a heterogeneous tumor (Sharpless and DePinho 2007; Gil et al., 2008). CSCs are believed to be highly tumorigenic and potentially metastatic, with resistance to most forms of radiation and chemotherapy (Nichols et al., 1998). Tumor growth is targeted by inhibiting DNA synthesis or cell division using anti-cancer drugs. Due to high clonogenic and tumorigenic capacity, some slow dividing CSCs protect themselves from therapy and lead to resistance (Yanamoto et al., 2014; Gil et al., 2008). Therefore failure of cancer treatment may be explained by improved understanding of the biological characteristics of CSCs. Identification of these CSCs markers may help in predicting therapeutic response and serve to optimize treatment plan to improve survival. 

Embryological stem cells have a central regulatory network that involves three master regulators for maintenance of the undifferentiated state. These include Oct4 (Pit Oct Unc [POU] domain transcription factor), Nanog (homeodomain transcription factor), and Sox-2 (high mobility group protein) (Nichols et al., 1998; Pesce et al., 1998; Mitsui et al., 2003). Additionally, Oct-4 and Nanog are purposed to be two of the four major factors that allow reprogramming of differentiated cells into pluripotent cells. Abnormal expression of these factors in stem cell and tumor tissues might play a vital role in tumor transformation, tumorigenicity, and tumor metastasis (Nichols et al., 1998; Pesce et al., 1998; Mitsui et al., 2003). 

CD24 is 27–amino-acid single-chain protein that is heavily O-and N-glycosylated glycosylphosphatidylinositol (GPI) - linked cell surface protein (Koukourakis et al., 2012; Kristiansen et al., 2003a). CD24 is a B-cell specific marker expressed in the early stages of B-cell development and also expressed in developing or regenerating tissue (Kristiansen et al., 2003a; Choi et al., 2007). P- Selectin is its only ligand identified till date (Koukourakis et al., 2012; Sung et al., 2010; Chou et al., 2007). CD24 functions as ligand to P-selectin, by virtue of which it facilitates interaction with platelets or endothelial cells, thereby increasing metastatic potential of tumour.* CD24 *expression has been identified as a prognostic marker in variety of tumors (Kristiansen et al., 2003a; 2003b; Sung et al., 2010; Choi et al., 2007; Chou et al., 2007; Sano et al., 2009)

Tsai et al showed increased expression of *Oct4* and *Nanog* correlated with a cisplatin-resistant phenotype as well as cancer recurrence while negatively correlated with differentiation status in OSCC (Tsai et al., 2011). In a study of Siu et al showed that embryonic stem cells express Oct4, which is lost upon differentiation. It was expressed in most invasive oral cancer cell lines indicating that Oct4 is a marker of invasiveness (Siu et al., 2012). Chiou et al., (2008) demonstrated that OSCC cases with expression of *Oct-4, Nanog,* and* CD133* had worst survival. It has also been shown that overexpression of Oct-4 and Nanog positively correlates with stage and chemo resistance, while negatively correlates with tumour grade. Habu et al., (2015) evaluated expression of *Oct4* and *Nanog* in 50 patients of HNSCC and suggested that these CSCs contribute significantly to the development of delayed neck metastasis by enhancing cell motility and invasiveness. In adenocarcinoma of lung, Oct4 and Nanog overexpression was associated with higher stage and shorter survival (Chiou et al., 2010). Hence, *Oct-4* and *Nanog* may acts as useful prognostic biomarkers for OSCC. 

Koukourakis et al., (2012) evaluated the role of CD24 and Oct4 in 74 locally advanced HNSCC and reported that extensive presence of Oct4 and CD24 was directly linked with increased proliferation index and poor prognosis. Kwon et al., (2007) analyzed the expression of *CD24* in 73 cases of uterine cervical SCC and found patients with negative CD24 having 20% less total failure and distant metastatic rates as compared to CD24 positive patients. The 5-year distant metastasis-free survival rate of CD24-negative patients was significantly greater than that of the CD24-positive patients (84.7% vs. 66.7%, respectively, p = 0.0497). Kristiansen et al., (2003a) evaluated *CD24* protein expression by immunohistochemistry in ovarian cancer and suggested a highly significant association of *CD24* overexpression with shortened patient survival. In addition, Kristiansen et al., (2003a) reported that *CD24* expression was associated with shortened disease free survival in breast cancer patients. Sung et al., (2010) evaluated the *CD24* expression of 140 patients with cervical SCC treated with chemo-radiotherapy after radical hysterectomy and concluded that *CD24 *expression was significantly associated with loco regional failure-free survival, distant metastasis-free survival and overall survival. Choi et al., (2007) reported that a *CD24* overexpression and loss of apical localization strongly predicts high tumor grade and stromal invasion in patients of urethral carcinoma. 

We have also observed statistically significant association between negative expression (<10%) of *Oct4, Nanog* and *CD24* and clinical or pathological response, there by indicating the possible potential of these markers as predictors for evaluating response to neoadjuvant chemo-radiotherapy in locally advanced OSCC.

We further observed that in advanced ypN and ypTNM stage, overexpression of *Oct4, Nanog* and *CD24* have a significant negative impact on survival outcomes. Moreover, patients exhibiting complete and partial response (clinically) or complete (RG1) and near-complete response (RG2) to neoadjuvant therapy show improved overall survival and recurrence free survival as compared to those with non-responders (clinically) or patients with residual tumor (RG3/RG4 pathologically) respectively. These findings are supported by previous reports (Driemel et al., 2009). Nevertheless, our study has few limitations. The sample size of study is small and the study design is retrospective. This may have introduced a selection bias. However, our inclusion criteria were stringent and our study protocol was strictly followed.

Currently, no single biomarker has been approved to accurately define CSCs in OSCC. However, a set of markers may help to target CSC population and identify patients with poor prognosis. This study may open new avenues for in-depth analysis and validation of the stem cell related genes studied herein as a useful predictive markers of response to the neoadjuvant therapy in OSCC patients. Further studies may be planned to focus to detail altered molecular pathways involved in resistance. The expression of these markers needs prospective validation to further elucidate their role as a predictive biomarkers for chemo-radiation response. 

In conclusion, our results suggest that expression of biomarkers *Oct4, Nanog* and *CD24* have a significant impact on treatment response and survival in patients with locally advanced OSCC treated with neoadjuvant chemo-radiation. Survival of these patients is significantly affected by ypN stage, ypTNM stage, expression of all three biomarkers, clinical and pathological response to neoadjuvant therapy.

## Funding

The study was partially funded by intramural research grant no, Dr. Ram Manohar Lohia Institute of Medical Sciences, Lucknow-226010, Uttar Pradesh, India.

## Conflict of interest

All authors have stated explicitly that there is no conflict of interest in connection with this article.
